# Physiological sensing systems and AI-based signal analysis in immersive virtual reality learning and training: A scoping review

**DOI:** 10.1371/journal.pone.0355596

**Published:** 2026-08-12

**Authors:** Wenxiang Zhou, Xiao Hu, Jeremy Tzi Dong Ng, Wei Wei

**Affiliations:** 1 Faculty of Applied Sciences, Macao Polytechnic University, Macao, China; 2 Faculty of Humanities and Arts, Hunan International Economics University, Changsha, China; 3 College of Information Science, The University of Arizona, Tucson, Arizona, United States of America; 4 Faculty of Education, The University of Hong Kong, Hong Kong SAR, China; Università degli Studi di Napoli Federico II, ITALY

## Abstract

This scoping review examined research on physiological sensing systems and AI-based signal analysis in immersive virtual reality (IVR) learning. It compared studies in terms of physiological modalities, HMD integration, calibration and synchronization, artifact management, feature engineering and representation learning, fusion strategies, validation design, and deployment readiness. We searched for studies published between 2016 and 2026 and included 19 empirical studies. In the 19 included studies, eye-tracking data (ETD, 13/19) and central nervous system signals (CNS, 11/19) were the most frequently reported modalities. CNS + ETD was the most frequently reported dual-modality configuration. Peripheral physiological signals (PPS, 3/19) were less frequently represented, and studies using electrodermal activity (EDA) were especially limited within the included corpus. The included studies covered the main stages of acquisition, preprocessing, feature engineering and representation learning, fusion, and analysis and modeling. Their targets included cognitive states, affective states, performance, attributes, and response patterns. Within this corpus, early fusion and traditional machine learning were frequently reported approaches. Reporting on sample-level processing, label construction, and train-test boundaries was incomplete in several studies. As a result, the interpretation and generalizability of reported findings depend on the specific study context. Overall, the included studies collectively outline an initial technical pathway, but evidence for reproducible, verifiable, and deployment-oriented real-time closed-loop IVR physiological sensing systems remained limited.

## Introduction

Immersive virtual reality (IVR) is increasingly used in education and training. It provides a strong sense of presence, three-dimensional interaction, and controllable scenarios. Because of these features, it is widely used in experimental teaching, skills training, and complex task practice [1–4]. Compared with desktop virtual environments, IVR links learning tasks, bodily actions, and scene feedback more closely [5,6]. However, most studies still focus on learning outcomes or experience design. A key issue for adaptive applications is how to continuously and objectively identify participants’ states, attributes, and response patterns during interaction [7,8].

Traditional assessment mainly relies on questionnaires, tests, or post hoc interviews. These methods are not well suited to capturing rapid changes in cognitive load, attention allocation, and emotional arousal during IVR learning [9–11]. Physiological signals provide a more continuous and objective source of information. Signals such as EEG, heart rate or pulse, skin conductance, and eye movement can reflect cognitive and affective states during task performance [12–14]. However, in IVR, these signals are not collected under stable and static conditions. They are affected by head movement, device wearing, tracking loss, trigger alignment, and multimodal interaction. Therefore, the key issue is not only whether AI can classify these signals. It is also how the sensing system is implemented, how the signals are cleaned and synchronized, and how the results are validated [15].

For this reason, research on AI-based physiological signal analysis in IVR learning should not be judged only by model performance. It should also be understood through the sensing system that supports it. The front-end includes sensor integration with head-mounted displays (HMDs), calibration, and synchronization. The middle stage includes artifact management, feature engineering, representation learning, and multimodal fusion. The back-end includes label construction, data splitting, generalization testing, and deployment readiness. Existing studies have discussed VR educational applications, physiological measurement, or AI methods separately [15–17]. However, studies that focus on IVR learning and training and compare these stages within one review framework are still limited. Therefore, this scoping review maps recent research on physiological sensing systems and AI-based signal analysis in IVR learning.

### Immersive Virtual Reality (IVR)

The core of IVR is not only virtual content itself. It is the tightly linked perception-action loop created by HMDs, spatial tracking, and multi-channel interaction [18–21]. This allows IVR to support high-risk and hard-to-reproduce experiments, as well as scenario-based training. It also creates opportunities for state sensing, performance evaluation, and dynamic adjustment during learning [22–24]. From a sensing perspective, however, these advantages also bring more complex system requirements. Learning tasks, body movements, and virtual scenes happen at the same time. As a result, sensors need to record continuously while still maintaining comfort and stability [18–24].

For this reason, studies on physiological signal-based identification and modeling in IVR learning should report how the sensing system was implemented. They should not focus only on the final algorithmic results [15–17].

### Physiological signals

In IVR learning research, common physiological signals can be grouped into three categories. The first is central nervous system signals, which reflect brain activity, such as EEG and fNIRS. The second is peripheral physiological signals and derived indicators, which reflect autonomic activity, such as PPG/rPPG, HR/HRV, and GSR. The third is eye-tracking data, which reflect visual attention and information processing paths [25–31]. These three categories provide different types of information about cognitive processing, arousal and emotion, and visual behavior [25–31]. Compared with self-report, they are better suited to capturing dynamic changes during IVR interaction. However, they are also affected by motion artifacts, contact quality, sampling stability, and the alignment of scene events [32–36].

Therefore, the value of physiological signals in IVR research depends not only on what is measured. It also depends on how signals are measured, how they are synchronized with the task, and how raw signals are turned into reliable analytical results [32–36].

### AI for physiological signal analysis in IVR learning: Role and constraints

AI, especially machine learning and deep learning, provides useful tools for extracting information from complex physiological signals in learning and training processes [37–41]. This information may relate to states, attributes, performance, and response patterns. However, under IVR conditions, the meaning of model outputs still depends strongly on the stability of front-end sensing, the quality of artifact management, multimodal alignment, and clear train-test boundaries [42–48]. In other words, even when studies report similar classification accuracy, differences in HMD integration, synchronization strategy, or train-test split strategy can affect how the results should be interpreted and how well they may transfer to other settings.

This suggests that AI-based physiological signal research in IVR learning is fundamentally a systems issue. The reliability of the results depends on sensing system implementation, signal processing, and validation design together. It does not depend only on back-end classifier comparison [42–48].

Existing reviews in related fields mainly follow three lines. One group focuses on the overall picture of IVR or VR educational applications [2,4,15,49]. Another group discusses wearables, physiological sensing, or AI analysis in education [7,12,17,50]. A third group focuses on physiological measurement and wearable biosensing in immersive experiences [8,51]. These reviews provide important background. However, several issues are still not well connected. These issues include which physiological modalities are used in IVR learning and training, how these modalities are integrated with HMDs and task events, how signals are cleaned, represented, fused, and modeled, and how much reported model performance is affected by label construction and train-test split strategies.

To show how this review differs from earlier work, Table 1 summarizes the focuses and boundaries of representative reviews. Compared with recent reviews that are closer to this topic [12,50,51], this review does not focus mainly on wearable physiological data and AI in general educational settings. Instead, it focuses on IVR learning and training. It compares studies in terms of HMD integration, calibration and synchronization, artifact management, feature engineering and representation learning, fusion strategy, label source, train-test split strategy, potential data leakage risk, and deployment readiness.

**Table 1:** Comparison of representative related reviews and the positioning of this study.

Review	Main focus	IVR focus	Sensing and AI pipeline	Distinct contribution
Radianti et al. [15] (2020)	IVR applications in higher education, design elements, and learning theories	Yes	No	Provides an overall educational landscape but does not discuss physiological sensing or AI-based signal analysis
Checa & Bustillo [49] (2020)	Learning/training applications of IVR serious games	Yes	No	Focuses on application forms without discussing physiological measurement or processing pipelines
Guillen-Sanz et al. [8] (2024)	Wearable biosensing in iVR experiences	Partly	Partly	Covers biosensors and HMD use, but is not centered on learning contexts and does not systematically review AI analysis workflows
Hong et al. [12] (2025)	Wearable sensors in learning analytics	No	Partly	A broad review of wearable sensors and learning analytics; not focused on IVR and does not compare sensing pipelines and validation boundaries study by study
Meini et al. [50] (2025)	Wearable biometrics and AI in education	No	Partly	Closest to the present work, but targets education in general rather than IVR; does not systematically review HMD integration, calibration/synchronization, artifact management, train-test splits, leakage risk, or deployment status
Bosta & Vosinakis [51] (2026)	Physiological measures for assessing immersive experience	No	Partly	Closest in topic, but not limited to IVR learning/training and does not systematically review the full pipeline
This study	Physiological sensing systems and AI-based signal analysis in IVR learning/training	Yes	Yes	Focuses on IVR and integrates evidence across sensing systems, signal processing, validation, and deployment

Based on these differences, this study uses a scoping review to map research on physiological sensing systems and AI-based signal analysis in IVR learning and training since 2016. This period is important because it marks a key stage in the commercialization of VR hardware. The review focuses on IVR settings. It uses sensing system implementation, signal processing, and validation as its main analytical lines. More specifically, it examines how physiological sensing systems were built, how signals were cleaned and represented, how multimodal data were fused, how models were validated, and how these studies were used to analyze and model states, attributes, performance, and related response patterns. It also examines how close these studies are to near-real-time or real-time closed-loop deployment. Based on this scope, the review addresses the following research questions:

RQ1: Which physiological signals were collected in IVR learning environments?
RQ2: How was AI used to analyze these physiological signals?
  RQ2a: Which AI techniques and methods were used?
  RQ2b: At which stages of the sensing and analysis pipeline were these AI techniques applied, such as acquisition, calibration and synchronization, preprocessing and artifact management, feature engineering, representation learning, fusion strategy, validation design, and deployment?

By addressing these questions, this review aims to provide two types of guidance for future research. First, it may help researchers choose appropriate physiological modalities, quality-control strategies, and AI methods. Second, it may provide more specific guidance for system design, including HMD integration, calibration and synchronization, label construction, participant-level validation, leakage control, and deployment readiness.

## Materials and methods

This study used a scoping review design. Its aim was to map the overall landscape of physiological signal research in immersive virtual reality (IVR) learning from the perspectives of sensing system implementation, signal processing, and AI modeling [52,53]. The methodological framework followed the five-stage approach proposed by Arksey and O’Malley and later guidance for scoping reviews [52,53]. In reporting, this study organized the research questions, information sources, screening process, data extraction, and presentation of results according to the core items of PRISMA-ScR [54] (S1 Checklist). No public protocol was prospectively registered for this review. The study was designed as an exploratory scoping review to map technical configurations, reporting practices, and evidence gaps in an emerging interdisciplinary area, rather than to estimate intervention effects or conduct a quantitative synthesis. Before screening began, the review questions, eligibility criteria, information sources, search strategy, and data-extraction framework were defined. These procedures were then applied consistently during title, abstract, and full-text screening, as well as during data extraction. This study also followed the transparency principles emphasized in PRISMA 2020 to improve reproducibility and reporting consistency [55]. The aim of this review was to map the evidence, compare technical pathways, and identify knowledge gaps. It was not intended to pool effect sizes. Therefore, no quantitative meta-analysis or formal risk-of-bias assessment was conducted.

A scoping review is suitable for clarifying conceptual boundaries, mapping technical routes, comparing study implementation, and identifying knowledge gaps [53]. Therefore, this review did not conduct quantitative synthesis. Instead, it organized the included studies around the two research questions: what physiological signals were collected, and how AI was used in the sensing-analysis-validation pipeline. The studies were compared in terms of physiological modalities, front-end acquisition, calibration and synchronization, preprocessing and artifact management, feature engineering, representation learning, fusion strategies, model families, validation design, and deployment status. To avoid conceptual confusion, SMOTE, oversampling, undersampling, and data augmentation were grouped as sample-level processing. They were reported separately and were not treated as signal quality control. For supervised or regression tasks, label sources, train-test split strategies, and potential data leakage risk were also recorded. This helped support the interpretation of model outputs and transferability.

### Inclusion and exclusion criteria

The inclusion criteria were as follows: (1) publication date between January 1, 2016, and March 29, 2026. The year 2016 was chosen as the starting point because commercial HMDs such as the Oculus Rift and HTC Vive entered the mainstream around this time, marking a period of rapid growth in immersive virtual reality research [56]. (2) The study should be empirical and should have collected at least one physiological signal in an immersive virtual reality learning or training context. Collection could be through wearable devices or through a clearly described and validated contactless approach. (3) AI techniques were actually used in the physiological signal analysis pipeline, including but not limited to machine learning classification, regression, clustering, deep learning-based representation learning, or fusion modeling. For studies that combined IVR physiological data collection with external-dataset model benchmarking, eligibility was based on whether the AI-based physiological analysis was explicitly developed as part of the IVR learning study; the use of an external benchmark dataset was recorded separately. (4) Eligible document types included peer-reviewed journal articles, conference papers, and full-text theses or studies available in institutional repositories. Because this topic is an emerging intersection of sensing, learning analytics, and VR, some technical implementations first appear in conference papers, theses, or institutional repository outputs. These sources were therefore retained to avoid missing early evidence [52–54]. However, publication type was treated only as a descriptive variable for evidence source and was not used as a direct proxy for methodological quality. No language filter was applied during database searching; however, only reports available in English were eligible at the screening stage. The exclusion criteria were as follows: (1) the study was not set in an IVR learning/training context; (2) it only discussed AR, MR, desktop VR, or other non-immersive environments; (3) it collected behavioral data only and no physiological signals; (4) it used AI but not for physiological signal analysis; or (5) the full text could not be obtained.

### Information sources and search strategy

Seven database searches were conducted: Web of Science Core Collection, Scopus, IEEE Xplore Digital Library, ACM Digital Library, SAGE Journals, ProQuest Dissertations & Theses Global, and a combined EBSCOhost search of Education Source and ERIC. Searches were conducted up to March 29, 2026. To balance sensitivity and topical focus, a two-stage search strategy was used. In the first stage, search strings were built around three core concepts—immersive virtual reality, physiological signals, and education/training contexts—and adapted to the fields and syntax of each database. The core search string was as follows: [(‘virtual reality’ OR ‘virtual environment’ OR ‘immersive virtual reality’ OR ‘immersive environment’ OR ‘VR’ OR ‘IVR’) AND (‘physiological signal’ OR ‘EEG’ OR ‘ECG’ OR ‘EDA’ OR ‘GSR’ OR ‘HR’ OR ‘HRV’ OR ‘PPG’ OR ‘EMG’ OR ‘RSP’ OR ‘Eye* track*’ OR ‘eye movement’) AND (‘education’ OR ‘educational’ OR ‘learner*’ OR ‘student*’ OR ‘teacher’ OR ‘instructor’ OR ‘learning’ OR ‘training’)]. The complete database-specific search strategies, including database platforms and collections, fields searched, limits, search dates, records retrieved, and record-management procedures, are provided in S2 Table.

AI-related keywords were not used as mandatory terms in the first-stage search because terminology in this field is highly heterogeneous. Some studies use broad labels such as machine learning or deep learning, some report only specific model names such as SVM, RF, CNN, or LSTM, and others refer only to tasks such as classification, prediction, or recognition in the methods or results. Adding AI terms too early could therefore exclude relevant studies whose terminology is scattered. For this reason, the first stage aimed to retrieve physiological measurement studies in IVR learning/training as completely as possible, and the second stage then determined at the full-text level whether AI was actually used for physiological signal analysis. This helped balance sensitivity and specificity. Study screening was conducted independently by two reviewers. The title, abstract, and full text of each record were assessed independently by at least two researchers. When disagreements occurred, two additional authors reviewed the record and consensus was reached through discussion.

### Study selection process

Study selection consisted of three levels: deduplication/initial filtering, title and abstract screening, and full-text assessment. Following the predefined strategy, 3650 records were identified across the seven databases. After database deduplication and an initial screening of document types, 1932 candidate records remained. A progressive title-abstract-full-text screening procedure was then applied. This left 521 records after title screening and 116 records for full-text assessment after abstract screening.

During full-text assessment, 15 studies that were not conducted in an immersive virtual reality learning or training context were excluded, as were 2 studies whose full texts could not be obtained. After this stage, 99 studies directly related to physiological signal measurement in IVR learning/training settings remained.

**Fig 1:**
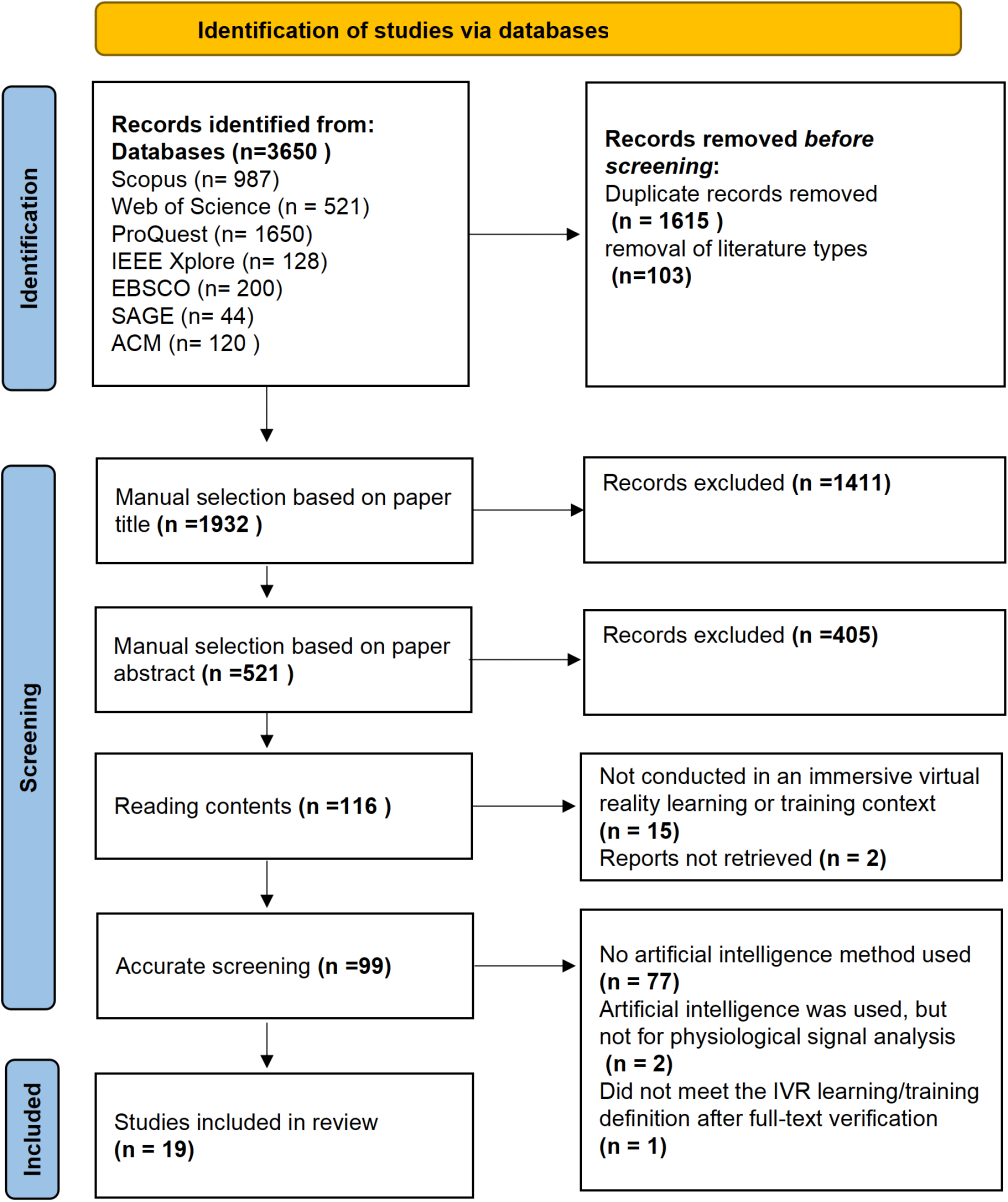
Study selection process (PRISMA-ScR).

In the second stage, these 99 full-text studies were further assessed to determine how AI was actually used, and the fit with the IVR learning/training definition was checked again. At this step, 77 studies that did not use AI, 2 studies that used AI but not for physiological signal analysis, and 1 study that did not meet the IVR learning/training definition after full-text review were excluded. This left 19 included studies. Fig 1 shows the screening process. The reports not retrieved and the reports excluded after full-text assessment, together with the primary reason for each decision, are listed in S2 Table.

### Data extraction and organization framework

Based on the research questions and the framework shown in Table 2, we extracted information on physiological signal types, front-end acquisition/calibration/synchronization, device models, sampling rates, preprocessing/artifact management, feature engineering, representation learning, fusion strategies, model families, AI task types, target constructs, and sample size. Where a study used an external benchmark dataset, the IVR sample and the external benchmark sample were recorded separately when identifiable. We also recorded evidence source, label source, train-test split strategy, potential data leakage risk, deployment status, and interpretability reporting to compare the interpretability, transferability, and practical feasibility of the reported systems. Processing steps such as SMOTE, oversampling, undersampling, and data augmentation were coded separately as sample-level processing and were not treated as signal quality control.

**Table 2:** Data extraction and organization framework.

Dimension	Subcategory	Description or examples
Physiological signal types (RQ1)	Central nervous system (CNS)	EEG and other CNS signals reported in the study (e.g., fNIRS)
	Peripheral physiological signals (PPS)	Cardiovascular sensing sources and derived indicators (PPG/rPPG; HR/HRV), EDA/GSR, EMG, and other peripheral physiological signals
	Eye-tracking data (ETD)	Eye movement/gaze/eye-movement trajectory data
Sensing and analysis pipeline (RQ2)	Acquisition/calibration/synchronization	Sensor-HMD integration, device model, sampling rate, pre-session calibration, trigger alignment, timestamp synchronization, and other explicitly reported front-end acquisition details
	Preprocessing/artifact management	Filtering, ICA, smoothing, interpolation, baseline correction, missing-value handling, invalid-sample removal, normalization, etc.
	Feature engineering	Handcrafted statistics, spectral and spatial features, CSP/FBCSP, gaze-object relative features, tsfresh/FRESH, MRMR, PCA, and related engineered features
	Representation learning	CNN, RNN/LSTM/GRU, CNN-LSTM, BERT, autoencoders, or other representations learned from raw or lightly processed signals
	Fusion strategy	No fusion/early fusion/intermediate fusion/late fusion/hybrid fusion
Model family (RQ2)	Traditional ML (Machine learning)	SVM, random forests, KNN, logistic regression, decision trees, XGBoost, naive Bayes, shallow ANN/MLP, etc.
	DL (Deep learning)	DNN, CNN, RNN/LSTM, Transformer, autoencoders, GANs, and other deep-network-centered models
	ML and DL reported	Studies reporting both traditional machine-learning and deep-learning models, whether the models were compared separately or combined within a single analytical pipeline
AI task type (RQ2)	Classification	Assigning physiological states, attributes, or groups to discrete categories
	Regression	Estimating continuous values such as attention or performance scores
	Clustering	Grouping similar physiological patterns under unlabeled conditions
	Prediction	Predicting learning outcomes, performance levels, or future physiological states
	Anomaly detection	Detecting fatigue, disengagement, or system faults
Target constructs, labels, and validation	Target constructs and label sources	Target constructs (e.g., immersion/distraction/learning performance/user identity) and label sources: self-report/task conditions/performance scores/expert ratings/participant attributes/physiological proxy indicators/unlabeled/not reported
	Train-test split strategy	Sample-level/trial-level/participant-level/session-level/external test/not reported; if a study explicitly reported multiple validation designs, they were recorded in parallel
	Potential data leakage risk	Low/high/not reported/Scheme-dependent/not applicable; if repeated windows, resampling, or data augmentation may cross split boundaries and participant/session separation is not explicit, the study was coded as high risk. Scheme-dependent was used when a study reported multiple validation schemes with different implications for data leakage risk
Dataset construction notes	Sample-level processing	SMOTE, oversampling, undersampling, and data augmentation are recorded separately and are not treated as signal quality control
Evidence source background	Journal/conference/dissertation/institutional repository	Used to describe evidence sources in a layered way; publication type is not treated as a proxy for methodological quality
Deployment and interpretability	Deployment status	Offline analysis/near-real-time analysis/real-time closed-loop deployment
	Interpretability reporting	Whether feature importance, SHAP/LIME, or other interpretability methods were reported
Sample size		Number of participants; IVR-study and external benchmark samples were distinguished where applicable

In addition, the included studies were organized at two levels. The first was the sensor/system level, including IVR scenario type, sensing implementation, integration with HMDs and tasks, device model, sampling rate, whether calibration or synchronization was reported, and the main artifact management steps. The second was the validation/deployment level, including target constructs, label sources, train-test split strategies, potential data leakage risk, deployment status, and interpretability reporting. In Table 3, “signal configuration” was coded only according to the physiological modalities included in each study. Auxiliary non-physiological features such as head/hand tracking, controller/posture data, text semantics, and clickstream data were not counted in the row labels and were instead described separately in the “System and validation notes” column of Table 4. When a study reported more than one AI task or more than one explicit validation design, these were recorded in parallel. As a result, some counts in Table 3 and S1 Table may exceed the total number of studies. Train-test split strategy and data leakage risk were judged only from information clearly reported or directly identifiable in the original text. When it was not possible to confirm whether train-test boundaries were separated across participants or sessions, the study was coded as “not reported.”

**Table 3:** Study counts and method distributions by signal configuration.

Signal configuration	No. of studies	Model family (traditional machine learning only/deep learning only/ML and DL reported)	Fusion strategy (no fusion/early/late)	Task type (classification/regression/clustering/prediction)
CNS	4	3/1/0	4/0/0	4/0/0/0
ETD	6	4/0/2	1/4/1	6/0/0/1
PPS	1	1/0/0	0/1/0	0/0/1/0
CNS + ETD	6	3/0/3	2/4/0	5/1/0/0
CNS + PPS	1	1/0/0	0/1/0	1/0/0/0
ETD + PPS	1	1/0/0	0/1/0	1/0/0/0

Model family is reported in the order traditional machine learning only/deep learning only/ML and DL reported; fusion strategy in the order no fusion/early/late, with intermediate fusion and hybrid fusion omitted because both were 0 in the included studies; task type in the order classification/regression/clustering/prediction, with anomaly detection omitted because it was 0 in the included studies. Signal configuration is coded only by physiological modalities; auxiliary non-physiological features such as head/hand trajectories, controller/posture data, text semantics, and clickstream data are not included in the row labels and are instead described in the “System and validation notes” column of Table 4. A study may contribute to more than one task type, so the corresponding counts may exceed the number of studies. Fusion strategy was coded according to the inputs jointly entered into the analytical model; fusion with auxiliary non-physiological features did not change the physiological signal-configuration category.

**Table 4:** Overview of physiological sensing systems, processing pipelines, and validation in the included studies.

Ref	IVR scenario	Physiological modality/device	Main processing pipeline	System and validation notes
[57]	Contextual English teaching	EEG (OpenBCI; 1000 Hz)	1–45 Hz band-pass + ICA; CNN and RNN-GRU learned frequency/time representations	Single modality; IVR EEG was collected and described as input to the CNN–RNN pipeline; comparative model performance was benchmarked on the external SEED-IV dataset; calibration/synchronization not explicitly reported; offline analysis
[58]	VR/online learning immersion evaluation	EEG + PPG (HR extracted; Pico Neo 2 + BrainLink + KS-CM01; sampling rate not reported)	Synchronized EEG/PPG acquisition; preprocessing and representation learning not reported	Early fusion of dual physiological modalities (EEG + PPG); HR extracted from PPG; k-fold tuning + independent test
[59]	VR driving learning transfer	ETD + heartbeat sensor (HR; HTC Vive Pro Eye 50 Hz + heartbeat sensor 500 Hz)	Pre-recording check and baseline; PCA dimensionality reduction	Dual physiological modalities; recorded in parallel with the task; train-test boundary not explicit
[60]	Internal/external distraction recognition in educational VR	EEG + ETD (device/sampling rate incompletely reported)	ICA + Z-score; SMOTE + undersampling; 1D CNN-LSTM/2D CNN representation learning	Early fusion of dual physiological modalities; cross-participant and cross-session tests; SHAP-based feature interpretation; offline classification
[61]	Distracted student detection in educational VR	EEG + ETD (OpenBCI + HTC Vive Pro Eye; EEG 125 Hz/eye tracking 120 Hz)	Eye-tracking calibration + EEG check; ICA; SMOTE + undersampling; CNN-LSTM representation learning	Early fusion of dual physiological modalities; offline classification; train-test boundary unclear
[62]	Teacher expertise recognition in a VR classroom	ETD (device not fully reported)	Preprocessing and feature-learning details not reported	Fused sensing-data modeling + interpretability analysis; offline identification; limited reporting on configuration/synchronization
[63]	Internal distraction detection in educational VR	EEG (OpenBCI; 125 Hz)	Electrode check + task-window alignment; ICA + Z-score; SMOTE + undersampling	Single modality; participant-level validation; offline classification
[64]	Attention and performance evaluation in remote learning	EEG + ETD (device/sampling rate not reported)	Independent analysis of two signals; FBCSP feature engineering	No feature-level fusion; offline evaluation; device/synchronization not reported
[65]	Immersive learning evaluation for university students	EEG	Preprocessing and feature engineering not reported	State differences induced by high- vs low-immersion conditions; offline classification; device/synchronization not reported
[66]	Dyslexia analysis in a VR reading task	ETD (device not reported)	Min-Max normalization; rotation augmentation; CNN + BERT representation learning	Late fusion with text semantics; offline diagnosis; augmentation and train-test boundary not clearly reported
[67]	Biomarker recognition of VR immersion	EEG (Meta Quest Pro + Waveguard 64/TMSi REFA-8; 2048 Hz)	Baseline calibration stage; ASA artifact removal + 0.1–30 Hz; MRMR selection	Single modality; 4-s windows; 80/20 train-test split; offline classification
[68]	Clustering cognitive/physiological responses in VR training	Video-derived rPPG (wearable comparator included)	Manual time-window features; clustering after PCA/t-SNE/UMAP	Unsupervised analysis; joint use of video physiology and motion signals
[69]	IVR learning-performance evaluation	ETD (HTC Vive Pro Eye; 120 Hz)	Task-synchronized recording; FRESH/tsfresh feature engineering	Early fusion of ETD with auxiliary head-pose and controller/interaction features; performance measures were used to construct outcome labels; 10-fold cross-validation
[27]	Continuous attention estimation in VR	EEG + ETD (BrainVision actiCHamp 32 channels + HTC Vive Pro Eye; eye/head 5 Hz)	Automatic synchronization of VR stimuli and EEG annotations; mean filtering; EEG feature imaging	Fusion with auxiliary features such as eye movements, head motion, and pupil diameter; leave-one-participant-out cross-validation
[30]	Measurement of attention, concentration, and distraction in VR	EEG + ETD	Preprocessing and representation-learning details not reported	Independent analysis of two signals; offline recognition; calibration/synchronization not reported
[70]	Distracted student detection in educational VR (eye tracking)	ETD (HTC Vive Pro Eye; 120 Hz)	Software calibration; invalid-sample removal + NaN + normalization; SMOTE/oversampling/undersampling; CNN/LSTM/CNN-LSTM with gaze/head features	Combined with auxiliary non-physiological features such as head orientation and scene-relative features; labels based on combined feedback/quiz rules
[71]	Gender differences in computational thinking learning in a VR classroom	ETD (HTC Vive Pro Eye/Tobii; 120 Hz)	Five-point calibration; low-tracking-rate sample filtering; smoothing/baseline correction/interpolation/I-VT; 43 eye-tracking features + SHAP	Single modality; participant-level nested 5-fold cross-validation
[72]	Learning-concentration recognition in VR	EEG + ETD (HTC Vive Pro Eye + Vive Face Tracker + ThinkGear/TGAM)	Device check + 5-min baseline; missing-value handling + standardization/normalization; 83 multimodal features	Joint use of auxiliary non-physiological features such as facial expressions, text, clickstream, and quizzes; 5-fold cross-validation
[73]	User identification in iVR serious games	ETD (HTC Vive Pro Eye; 120 Hz)	Pre-session calibration; outlier removal; FRESH/tsfresh features from eye/head/hand time series	Comparison of mixed-session and cross-session schemes; integrated head/hand tracking features; offline identification

In this table, the “Physiological modality/device” column encodes only the primary physiological modality. Auxiliary non-physiological input features such as head/hand trajectories, controller/posture data, text semantics, and clickstream data are described in the “System and validation notes” column. Variables used only to construct outcome labels are identified separately. PPG and rPPG denote sensing sources, whereas HR and HRV denote indicators derived from them; when both were reported, this review uses the format “sensing source (derived indicator).”

## Results

Nineteen studies were ultimately included. Table 3 summarizes method distributions under different signal configurations. Table 4 provides an overview of the physiological sensing systems, processing pipelines, and validation characteristics of the included studies. S1 Table summarizes target constructs, label sources, train-test split strategies, data leakage risk, and deployment status. The sections below further report front-end acquisition, preprocessing, feature/representation learning, fusion approaches, and validation and deployment characteristics.

### RQ1: Which physiological signals were collected in IVR learning environments?

Among the 19 included studies (Table 3), when each signal type was counted once per study, eye-tracking data (ETD) was the most widely used modality (13 studies, 68.4%), followed by central nervous system signals (CNS; 11 studies, 57.9%). Peripheral physiological signals (PPS) were used much less often (3 studies, 15.8%). In AI-supported immersive virtual reality (IVR) learning environments, no studies were found that mainly focused on common autonomic signals such as electrodermal activity (EDA). It is also notable that 8 of the 19 studies (42.1%) used combinations of signals. CNS + ETD was the most common combination (6 studies, 31.6%), whereas CNS + PPS and ETD + PPS each appeared in only 1 study. Overall, within the included studies, single-modality and dual-modality designs were more common than designs collecting all three physiological signal categories.

### RQ2: How was AI used in physiological signal analysis?

#### Front-end acquisition, calibration/synchronization, and preprocessing/artifact management.

Based on the reporting in the included studies, relatively few of the 19 papers clearly described front-end acquisition, calibration, or time synchronization details. Most studies stated that EEG, ETD, or PPS was used, but gave only limited information on sensor-HMD integration, pre-session calibration, trigger alignment, or modality synchronization. Clearer examples were mainly found in eye-tracking studies. For example, Miguel-Alonso et al. [73] reported eye-tracking calibration before each session. Some studies also mentioned synchronized recording with the task process or the exclusion of samples with low tracking rates. Overall, however, transparency in front-end implementation details remains limited. In contrast, preprocessing and artifact-management procedures were reported somewhat more often, and some modality-specific reporting patterns were observed in the included studies. EEG studies more often relied on ICA to remove ocular or muscle artifacts [57,60,61,63], whereas eye-tracking studies more often used smoothing, baseline correction, interpolation, invalid-sample removal, missing-value handling, and standardization to control data quality [70–73].

It is important to distinguish sample balancing or data augmentation from signal quality control. These procedures are better treated as sample-level processing at the dataset level rather than as signal cleaning itself. In the included studies, SMOTE or oversampling/undersampling appeared mainly in [60,61,63,70], while rotation-based augmentation appeared in [66]. These operations may improve class balance or enlarge the training set, but they should be separated from sensor noise suppression, artifact removal, or temporal synchronization. They are also closely related to later train-test boundaries. When data splitting is not clearly reported, such processing can add uncertainty to the interpretation of model performance.

#### Feature engineering and representation learning.

At the representation level, the included studies followed two parallel routes: manual feature engineering and deep representation learning. For EEG, methods such as CNN, CNN-LSTM, RNN/GRU, and EEG feature imaging were more often used to learn spatial-frequency-temporal representations directly from raw or lightly processed sequences [27,57,60,61]. In contrast, ETD single-modality studies more often relied on sliding-window statistical features, object/avatar-relative features, large-scale feature engineering with FRESH/tsfresh, and the use of SHAP at the back-end to interpret key features [69,71,73]. In addition, methods such as PCA, FBCSP, and MRMR are better understood as feature engineering or dimensionality-reduction/selection steps rather than as “AI preprocessing” in a broad sense. Making this distinction helps clarify which methods are used to clean signals, which ones are used to build interpretable features, and which onesb are used to learn latent representations.

#### Data fusion.

Overall, 12 of the 19 studies used fusion analysis (63.2%). Of these, 6 collected more than one physiological signal. Another 6 studies collected only one main physiological signal but also combined head/hand trajectories, controller/posture information, text semantics, or other interaction features to improve classification or clustering performance. In Table 3, these studies are still classified by their main physiological modality, while the auxiliary non-physiological features are described separately in Table 4. In addition, 2 studies collected two different physiological signals at the same time but did not integrate them at the feature level. Instead, learning outcomes were judged from the separate analyses of the two signals [30,64]. Under the early/intermediate/late classification framework in multimodal fusion research [74,75], early fusion was the most frequently reported fusion strategy among the 12 fusion studies (11/12), whereas late fusion appeared in 1 study and no included study reported intermediate or hybrid fusion. This suggests that current studies more often combine multi-source features directly at the input level, while discussion of representation-level or decision-level fusion remains limited. The reasons for this observation and its methodological implications are discussed further in the Discussion section.

#### Model families.

Across signal configurations, studies using only traditional machine learning were the largest group, with 13 studies (68.4%). The main algorithms were support vector machines (SVM), random forests (RF), and k-nearest neighbors (KNN). Studies reporting both traditional machine-learning and deep-learning models ranked second, with 5 studies (26.3%). Studies using only deep learning were the smallest group, with just 1 study (5.3%). Among the 11 single-modality studies, traditional machine learning alone accounted for 72.73% (8/11), studies reporting both ML and DL accounted for 18.18% (2/11), and deep learning alone accounted for 9.09% (1/11). Among the 8 multimodal studies, 5 used only traditional machine learning, while 3 reported both ML and DL, mainly in CNS + ETD studies. In the sample of this review, studies reporting both ML and DL were more frequently observed in multimodal studies than in single-modality studies, although this pattern should be interpreted cautiously because of the small subgroup sizes.

#### Task types.

In the included studies, classification was the most frequently reported AI task, appearing in 17 studies (89.5%). At the same time, regression, clustering, and prediction each appeared in 1 study, and anomaly detection did not appear in any study. It should be noted that one study could be counted in more than one task category, so the sum of task counts may exceed the total number of studies. For example, Serrano-Mamolar et al. [69] included both classification of learning-environment quality and prediction of learning performance, so prediction was not zero. For clustering, Walawwe and Ekanayake [68] used an unsupervised approach to group cognitive/physiological response patterns. For regression, Delvigne et al. [27] estimated attention levels in a continuous framework. Within this review sample, work on continuous state estimation, forward-looking prediction, and anomaly detection was less frequently represented.

#### Evidence sources, target constructs, label sources, train-test split strategies, data leakage risk, and deployment status.

In terms of evidence source, the 19 included studies were mainly peer-reviewed journal articles (11/19) and conference papers (6/19), with 1 thesis [30] and 1 institutional repository study [68]. At the same time, target constructs and label sources in supervised or regression tasks showed high heterogeneity. Some studies modeled states such as distraction, immersion, cognitive load, or learning concentration [58–61,63,65,67,70,72]. Some focused on learning performance or environment quality [64,69]. Others addressed emotional states, diagnosis, teacher expertise, gender attributes, or user identity [57,62,66,71,73]. Label sources included task conditions, performance outcomes, combinations of self-report and performance, expert judgement, participant attributes, and physiological proxy indicators. This means that “classification” did not always refer to the same type of problem across studies, and model performance should not be compared directly across studies. A summary is provided in S1 Table.

In terms of train-test partitioning, risks of data leakage and practical deployment feasibility, the majority of reviewed studies used offline laboratory analytical schemes. Some studies clearly used sample-level splits [57,58,67,69,72,73], participant-level splits [27,60,63,71], and session-level or cross-session schemes [60,73]. Because this review allowed multiple explicit validation designs to be recorded in parallel, the sum of these counts may exceed the total number of studies, but train-test boundaries were still not sufficiently reported in many other studies. If the one unsupervised clustering study is excluded, among the 18 supervised or regression studies, 4 were coded as low risk for data leakage, 5 as high risk, 8 as not reported, and 1 as scheme-dependent because it reported multiple validation schemes with different implications for data leakage risk. In this review sample, AI-based physiological signal analysis was mainly reported in offline discrimination settings, and evidence for real-time, continuous, closed-loop IVR adaptation under strict generalization testing was limited.

## Discussion

Taken together, the findings for RQ1 and RQ2 show that research in this field is shaped not only by whether physiological signals are collected, but also by how acquisition, cleaning, fusion, validation, and application are linked. Compared with reviews on wearables or AI in general educational settings, this review places more emphasis on sensing system implementation, signal processing, and validation design in IVR contexts. The discussion is organized around three aspects: the sensor layer, the signal-pipeline layer, and the reproducibility and deployment layer.

### Sensor layer: Coverage of wearable/embedded modalities, HMD integration, and scenario fit

In terms of modality coverage, ETD (13/19) and CNS (11/19) were the two most frequently reported sources in the included IVR learning studies. This pattern is closely related to hardware integration. Eye tracking is often built into the HMD or closely linked to head pose. This makes it suitable for capturing task-related information such as gaze allocation, object focus, and scene navigation [70,72,73]. By contrast, EEG has been used to infer cognitive states such as cognitive load, concentration, and distraction, but such inferences require caution in IVR because EEG recordings are sensitive to head movement, oculomotor activity, muscle activity, wearing stability, and electrode contact quality [57,60,63]. Therefore, although both eye tracking and EEG are used in IVR physiological sensing, they differ clearly in hardware burden, comfort, motion tolerance, construct interpretation, and the difficulty of HMD integration. Current studies still do not report these system-level differences in sufficient detail [57,60,63,70,72,73].

PPS appeared in only 3 included studies. These studies mainly used cardiovascular sensing sources and derived indicators such as PPG/rPPG and HR. As a result, emotional arousal, stress responses, and autonomic activity are still only lightly covered. In addition, existing multimodal studies are not the same as multi-physiological sensing systems. Besides dual physiological combinations, many studies combined one physiological signal with interaction data such as head and hand tracking, text semantics, clickstreams, or quiz responses [66,70,72,73]. This pattern may indicate an emerging interest in multi-source modeling within the included studies. However, more complete wearable or embedded physiological combinations are still uncommon. This is especially true for peripheral modalities such as EDA, which could add useful information about emotion and arousal. There are also still few systematic comparisons of comfort, synchronization cost, and robustness across modalities in HMD settings.

### Signal-pipeline layer: Calibration and synchronization, artifact management, and multimodal fusion

At the signal-pipeline level, the included studies already cover the main stages of acquisition, preprocessing, feature engineering and representation learning, fusion, and analysis and modeling. However, most studies report algorithmic results more clearly than the sensing pipeline behind them. In preprocessing, EEG studies often used ICA to remove ocular or muscle artifacts. By contrast, eye-tracking studies more often used smoothing, baseline correction, interpolation, invalid-sample removal, and standardization [57,60,63,71,73]. These steps suggest that researchers are aware of the effects of head movement, occlusion, tracking loss, and multi-device interference on signal quality in IVR. However, only a limited number of studies reported calibration and synchronization in a systematic way. A few studies provided clearer front-end details. For example, Abdurrahman et al. [59] reported 50 Hz eye tracking and 500 Hz heart-beat recording. Study [67] reported Meta Quest Pro with 2048 Hz EEG acquisition. Studies [71] and [73] reported Vive Pro Eye at 120 Hz and pre-session calibration. Study [27] described automatic annotation synchronization between VR stimuli and EEG. Even so, most studies gave only brief information on device models, sampling rates, timestamp alignment, pre-session calibration, and synchronization between sensors and HMD or task events. As a result, even when studies are grouped under labels such as “EEG+ETD” or “ETD+interaction features,” their acquisition conditions may still differ in important ways.

An additional issue concerns the construct validity of EEG-based cognitive-load inference in IVR. Frontal or midline theta has often been found to associate with working-memory load and task demand [76,77], but in IVR it should not be interpreted as a direct or stand-alone marker of cognitive load. Head movement, changes in electrode contact, ocular activity, and scalp or neck muscle activity may co-vary with task difficulty and interaction style [78]. Therefore, high classification accuracy alone is insufficient to establish that this EEG feature captures cognitive load. Future IVR studies should strengthen convergent validity through explicit EOG/head-motion/EMG artifact checks, task-load manipulations, behavioral performance and subjective workload measures, and participant- or session-level generalization tests, as well as combining EEG features with other modalities such as eye-tracking indicators. Under such convergent evidence, frontal theta may be treated as supportive evidence of cognitive-load-related processing; without it, EEG-based labels should be interpreted more conservatively as context-specific discriminative patterns.

In the included studies, feature-engineering, representation-learning, and fusion methods were often relatively simple to implement. EEG and EEG + ETD studies more often used deep models such as CNN, CNN-LSTM, or feature imaging [27,57,60,61]. By contrast, ETD single-modality studies more often used sliding-window statistical features, FRESH/tsfresh, or manual feature engineering [69,71,73]. At the fusion level, early fusion was the most frequently reported strategy among the 12 fusion studies. Late fusion appeared in only 1 study, and no study used intermediate fusion or hybrid fusion. Within this review sample, multi-source features were often combined directly at the input level. One reason may be the need to reduce system complexity in small-sample settings. However, this approach does not address cross-modal temporal alignment, representation learning, or uncertainty propagation in a systematic way. In addition, some studies collected two physiological signals at the same time but did not truly integrate them at the feature level [30,64]. This means that multimodal acquisition and multimodal fusion are not the same in the current literature.

In terms of validation design, label sources, and deployment readiness, the included evidence was concentrated mainly in offline classification studies. Studies using only traditional machine learning accounted for 13 of the 19 included studies. Classification appeared in 17 studies. Regression, clustering, and prediction each appeared only once. Anomaly detection did not appear in any study. In addition, label sources in supervised tasks were highly heterogeneous. Some labels were based on experimental task conditions. Others were based on scores or performance. Some came from self-report, expert or existing diagnosis, or participant attributes. A few were based on physiological proxy indicators. If the one unsupervised clustering study is excluded, among the 18 supervised or regression studies, 4 were coded as low risk for data leakage, 5 as high risk, 8 as not reported, and 1 as scheme-dependent. These findings suggest that, in this review sample, AI-based analysis was mainly used for offline discrimination of experimental data. Its use in real-time, continuous, closed-loop IVR adaptation under strict generalization testing is still limited.

### Reproducibility and deployment layer: From algorithmic experiments to deployable sensing systems

From the overall reporting pattern in the included studies, one recurring issue was not simply whether AI was used, but whether the sensing system was described clearly enough to support reproduction, comparison, and deployment. Most studies reported the use of EEG, ETD, or PPS. However, reporting was still uneven for device models, sampling rates, sensor placement, mechanical and logical integration with the HMD, calibration procedures, synchronization methods, validation design, and whether the analysis was offline, near-real-time, or real-time. This problem is especially clear in validation design. Some studies clearly reported sample-level, participant-level, or session-level splits [27,57,58,60,63,67,69,71–73]. However, in many other studies, the train-test boundaries were still not reported clearly enough. In this type of research, such information is essential for interpreting model performance. Only when the acquisition chain, processing chain, and validation chain are all described clearly can findings be compared more directly and moved closer to practical deployment.

Future research should treat signal-pipeline reproducibility and model performance as joint priorities. In addition to expanding PPS coverage and combining multiple physiological modalities, the field may benefit from a minimum reporting set. At a minimum, studies should report the HMD and sensor models, signal acquisition positions, sampling rates, calibration and synchronization methods, the main artifact-management steps, whether sample-level processing was applied only after data splitting, label sources, train-test split strategies, especially whether they were participant-level, session-level, or external-test designs, the stage at which fusion occurred, and whether the system supports near-real-time or real-time closed-loop operation.

## Conclusion

This review mapped research on AI-based physiological signal analysis in IVR learning environments since 2016. It examined this field through three main aspects: sensing system implementation, signal processing, and validation and deployment. The findings suggest that, in the included literature, progress in AI-based physiological signal analysis for IVR learning depends not only on model performance, but also on the quality of sensing-system reporting and the clarity of validation design.

Across the 19 included studies, eye-tracking data (ETD) and central nervous system signals (CNS) were the most common modalities. CNS + ETD was the most common dual-modality configuration. Peripheral physiological signals (PPS) were used much less often, especially autonomic indicators such as EDA. Existing studies already cover the main stages of acquisition, preprocessing, feature engineering and representation learning, fusion, and analysis and modeling. However, reporting on HMD integration, device models, sampling rates, and calibration and synchronization was still uneven. In addition, sample-level processing was not always clearly separated from signal quality control. Although a small number of studies used explicit sample-level, participant-level, or session-level validation designs, many studies still did not report label construction and train-test boundaries clearly enough. Thus, the generalizability of reported model performance is still difficult to assess.

Overall, the included studies collectively outline an initial technical pathway. However, within this review sample, evidence for reproducible, verifiable, and deployment-oriented real-time closed-loop IVR physiological sensing systems remained limited. Future research should expand PPS coverage, improve reporting on sensing system implementation and artifact management, use stricter validation designs, and further develop multimodal fusion, interpretability analysis, and near-real-time or real-time closed-loop IVR adaptive applications. Future research should also develop a task- and context-sensitive taxonomy of IVR learning and training scenarios and target constructs, so that generic labels such as distraction, attention, concentration, immersion, and cognitive load can be defined in relation to specific task demands, learner actions, experimental manipulations, and label sources, thereby improving cross-study comparability. Future deployment-oriented studies should also report practical system-level metrics for standalone HMD implementation, including whether physiological processing and model inference are performed on-device, in the cloud, or through a hybrid architecture, as well as privacy safeguards and end-to-end latency from signal acquisition to adaptive feedback.

## Limitations

Although this study met its main aims through a structured scoping review process, several limitations should be noted.

First, only 19 studies met the inclusion criteria. Therefore, the evidence base was still limited. This may affect the representativeness and generalizability of some findings in more specific areas. However, the small number of studies also reflects the fact that this topic is still a relatively new area at the intersection of several fields. The 19 included studies still provide an initial overview of several technical paths observed in the current literature, although this overview should not be considered exhaustive. In addition, this review used multiple databases and independent screening by two reviewers to improve the relevance, reliability, and representativeness of the included evidence. To avoid missing early technical work in this emerging field, a small number of theses and institutional repository studies were also included. Although these sources were described separately by evidence type, differences in maturity across publication types may still have introduced some heterogeneity.

Second, this review did not subdivide IVR learning scenarios, such as knowledge instruction and skills training, or learner characteristics, such as age and cognitive level. As a result, it could not show how different settings and populations may require different physiological sensing and AI analysis methods. In addition, practical issues such as cost-effectiveness and data privacy in real-world deployment were not examined in depth.

Third, a scoping review focuses on systematic mapping and trend identification rather than formal methodological quality assessment or quantitative synthesis. For this reason, it is difficult to quantify differences in the performance of different physiological signal types or AI algorithms. Although this review also recorded evidence sources, label sources, train-test split strategies, potential data leakage risk, and validation and deployment readiness, these dimensions were used only for descriptive comparison. They cannot replace formal quality appraisal in a systematic review.

Another limitation is related to the time window of the review. The starting year of 2016 was selected because commercial HMD-based IVR systems became more widely used in learning and training research since about that time [79]. However, some foundational work on physiological-signal calibration, synchronization, and artifact management had already been developed before 2016. Therefore, some earlier methodological reports with detailed guidance on signal quality control may not have been included. This time boundary should be considered when interpreting the findings. The included records were counted at the publication level rather than as confirmed independent datasets; therefore, potentially related or extended reports should be considered when interpreting study-frequency counts.

## Supporting information

S1 Checklist PRISMA-ScR checklist. Completed PRISMA-ScR (Preferred Reporting Items for Systematic Reviews and Meta-Analyses extension for Scoping Reviews) checklist, indicating the section and page of the manuscript where each item is reported.
(DOCX)

S1 Table Summary of validation information for the included studies. Target constructs, label sources, train-test split strategies, potential data leakage risk, and deployment status of the 19 included studies.
(DOCX)

S1 Data Study-level extracted and coded data underlying the descriptive synthesis. The dataset contains study characteristics, sample-size information, physiological modalities, sensing and processing information, model families, fusion strategies, AI task types, target constructs, label sources, validation designs, potential data leakage risk, interpretability reporting, and deployment status for the 19 included studies. IVR-study and external benchmark samples are distinguished where applicable.
(XLSX)

S2 Table Database-specific search strategies and full-text screening decisions. The file provides database platforms and collections, fields searched, exact database-specific search strategies, limits, search dates, records retrieved, record-management procedures, and the reports not retrieved or excluded after full-text assessment, with the primary reason for each decision.
(XLSX)
