# Supplementary material for: Physiological sensing systems and AI-based signal analysis in immersive virtual reality learning and training: A scoping review
Source: PLoS One. 2026 Aug 12;21(8):e0355596. doi: 10.1371/journal.pone.0355596 (PMC13465850; doi:10.1371/journal.pone.0355596)
Supplement: Supplementary file 2 [file pone.0355596.s002.docx]

**S1 Table. Summary of validation information for the included studies**

| **Ref** | **Evidence source** | **Target construct / label source** | **Train-test split strategy** | **Data leakage risk** | **Deployment status** |
| --- | --- | --- | --- | --- | --- |
| [57] | Journal | Target construct: emotional state Label source: not reported | Sample-level split | Not reported | Offline analysis |
| [58] | Journal | Target construct: learning immersion experience Label source: task condition | Sample-level split | Not reported | Offline analysis |
| [59] | Journal | Target construct: neurocognitive load / learning transfer Label source: task condition | Not reported | Not reported | Offline analysis |
| [60] | Journal | Target construct: internal/external distraction Label source: task condition | Participant-level split / session-level split | Low | Offline analysis |
| [61] | Conference | Target construct: distraction state Label source: task condition | Not reported | High | Offline analysis |
| [62] | Conference | Target construct: teacher expertise Label source: participant attributes / expert rating | Not reported | Not reported | Offline analysis |
| [63] | Conference | Target construct: internal distraction Label source: task condition | Participant-level split | Low | Offline analysis |
| [64] | Conference | Target construct: learning attention / learning effectiveness Label source: performance scores | Not reported | Not reported | Offline analysis |
| [65] | Conference | Target construct: learning immersion Label source: task condition | Not reported | Not reported | Offline analysis |
| [66] | Journal | Target construct: reading difficulty / dyslexia diagnosis Label source: expert rating | Not reported | High | Offline analysis |
| [67] | Journal | Target construct: immersion state Label source: task condition | Sample-level split | High | Offline analysis |
| [68] | Institutional repository | Target construct: cognitive/physiological response patterns Label source: unlabeled | Not applicable | Not applicable | Offline analysis |
| [69] | Journal | Target construct: learning environment quality / learning performance Label source: task condition / performance scores | Sample-level split | High | Offline analysis |
| [27] | Conference | Target construct: attention level Label source: performance scores | Participant-level split | Low | Offline analysis |
| [30] | Dissertation | Target construct: attention / concentration / distraction Label source: task condition | Not reported | Not reported | Offline analysis |
| [70] | Journal | Target construct: distraction level Label source: self-report / performance scores | Not reported | High | Offline analysis |
| [71] | Journal | Target construct: gender attribute Label source: participant attributes | Participant-level split | Low | Offline analysis |
| [72] | Journal | Target construct: learning concentration Label source: physiological proxy indicator | Sample-level split | Not reported | Offline analysis |
| [73] | Journal | Target construct: user identity Label source: participant attributes | Sample-level split / session-level split | Scheme-dependent | Offline analysis |

Note: S1 Table summarizes the target constructs, label sources, train-test split strategies, data leakage risk, and deployment status of the included studies. Label sources were coded only from information identifiable in the original text. If a study explicitly reported multiple validation designs, they were recorded in parallel, so some totals may exceed the number of studies. Train-test boundaries not reported were coded as "Not reported"; only explicitly participant-external, session-external, or external-test designs were coded as "Low" risk; studies using sliding windows, resampling, or augmentation without explicit cross-participant or cross-session separation were coded as "High"; "Scheme-dependent" was used when a study reported multiple validation schemes with different implications for data leakage risk; unsupervised clustering was coded as "Not applicable".
