# Supplementary material for: Physiological sensing systems and AI-based signal analysis in immersive virtual reality learning and training: A scoping review
Source: PLoS One. 2026 Aug 12;21(8):e0355596. doi: 10.1371/journal.pone.0355596 (PMC13465850; doi:10.1371/journal.pone.0355596)
Supplement: Supplementary file 3 [file pone.0355596.s006.docx]

**Original Article Title: “Physiological Sensing Systems and AI-Based Signal Analysis in Immersive Virtual Reality Learning and Training: A Scoping Review**”

**To:** PLOS ONE Editor

**Re:** Response to the Reviewers

Dear Editor,

We thank the editor and reviewers for the opportunity to revise our manuscript. The comments have helped us improve the clarity and methodological transparency of the paper.

In the revised manuscript, we have made changes in response to the reviewers’ suggestions. We clarified EEG-based cognitive-load inference in IVR, added discussion of convergent validity and artifact-related concerns, and revised the language throughout the manuscript to ensure claims remain appropriate to the scope of 19 included studies. We also clarified the rationale and limitation of the 2016–2026 search window, explained the absence of prospective registration, and confirmed that the review criteria were established prior to screening.

We further revised the Conclusion to include recommendations on future task/construct taxonomy and practical deployment metrics for standalone HMD systems. In addition, we checked the references, subsection numbering, table citations, author affiliation marks, and overall formatting, and reformatted the manuscript using the journal’s template.

A point-by-point response to the reviewers’ comments is provided below.

Best regards,

Xiao Hu

Associate Professor,

College of Information Science, The University of Arizona

Email: xiaohu@arizona.edu

**Reviewer #1, Comment #1:** EEG in IVR is prone to motion and oculomotor confounds. Does current literature adequately justify linking frontal theta to 'cognitive load'? Consider adding a short Discussion paragraph on convergent validity tasks beyond classification accuracy.

**Author response:** We appreciate your thoughtful comments. We agree that EEG recorded in IVR is vulnerable to motion, oculomotor, muscle-related, and electrode-contact confounds, and that frontal theta should not be interpreted as a stand-alone marker of cognitive load. We also recognize that, in the original version, although we discussed artifact management, synchronization, and validation boundaries, we did not explicitly address the construct-validity issue involved in linking EEG features, especially frontal theta, to cognitive load.

Following the reviewer’s suggestion, we added a paragraph to the Discussion on the construct validity of EEG-based cognitive-load inference in IVR, clarifying that frontal theta should be treated as supportive evidence only when adequate artifact control and convergent validation are in place. The wording in the sensor-layer discussion was also revised to describe EEG as a modality that may provide information related to cognitive load under appropriate control conditions, rather than as a direct source of cognitive-load measurement. In addition, convergent-validity recommendations were added beyond classification accuracy, covering task-load manipulation, behavioral performance, subjective workload measures, eye-tracking indicators, artifact-control checks, and generalization tests.

Revised text within the subsection titled *Sensor Layer: Coverage of Wearable/Embedded Modalities, HMD Integration, and Scenario Fit* in the Discussion section, lines 406–412.

"By contrast, EEG has been used to infer cognitive states such as cognitive load, concentration, and distraction, but such inferences require caution in IVR because EEG recordings are sensitive to head movement, oculomotor activity, muscle activity, wearing stability, and electrode contact quality [57, 60, 63]. Therefore, although both eye tracking and EEG are used in IVR physiological sensing, they differ clearly in hardware burden, comfort, motion tolerance, construct interpretation, and the difficulty of HMD integration."

We also added a short paragraph (lines 447–460) emphasizing that classification accuracy alone should be interpreted as evidence of discriminative performance, not as sufficient evidence of construct validity:

“An additional issue concerns the construct validity of EEG-based cognitive-load inference in IVR. Frontal or midline theta has often been found to associate with working-memory load and task demand [76, 77], but in IVR it should not be interpreted as a direct or stand-alone marker of cognitive load. Head movement, changes in electrode contact, ocular activity, and scalp or neck muscle activity may co-vary with task difficulty and interaction style [78]. Therefore, high classification accuracy alone is insufficient to establish that this EEG feature captures cognitive load. Future IVR studies should strengthen convergent validity through explicit EOG/head-motion/EMG artifact checks, task-load manipulations, behavioral performance and subjective workload measures, and participant- or session-level generalization tests, as well as combining EEG features with other modalities such as eye-tracking indicators. Under such convergent evidence, frontal theta may be treated as supportive evidence of cognitive-load-related processing; without it, EEG-based labels should be interpreted more conservatively as context-specific discriminative patterns.”

**Reviewer #1, Comment #2:** Foundational work on calibration/artefact management existed before 2016. Could your search window have systematically excluded older, more detailed reports? A brief acknowledgment in Limitations would add nuance.

**Author response:**

Thank you for the suggestion. Our review focused on studies published from 2016 onward because this period corresponds to the wider adoption of commercial HMD-based IVR systems and the rapid growth of IVR learning/training research using AI-based physiological signal analysis. However, we agree that this time window may have excluded older methodological reports before 2016 that may have provided more details on calibration, signal quality control, synchronization, and artifact management. We have therefore added a brief acknowledgment in the Limitations section (lines 577–584). The added text reads:

“Another limitation is related to the time window of the review. The starting year of 2016 was selected because commercial HMD-based IVR systems became more widely used in learning and training research since about that time [79]. However, some foundational work on physiological-signal calibration, synchronization, and artifact management had already been developed before 2016. Therefore, some earlier methodological reports with detailed guidance on signal quality control may not have been included. This time boundary should be considered when interpreting the findings.”

**Reviewer #1, Comment #3:** Although Limitations are well discussed. None of the 19 studies address on device vs. cloud privacy or latency on standalone HMDs. Would you recommend future work report such metrics? One sentence in Conclusion would increase practical impact.

**Author response:** Thanks for the suggestion. We added one sentence to the Conclusion section to explicitly highlight that future deployment-oriented studies should report these practical system-level considerations including processing locations (on-device vs. cloud), privacy safeguards, and end-to-end latency. This addition improves the practical relevance without expanding the scope of the review beyond the evidence available from the 19 included studies. The supplementary text appears in lines 544–548:

“Future deployment-oriented studies should also report practical system-level metrics for standalone HMD implementation, including whether physiological processing and model inference are performed on-device, in the cloud, or through a hybrid architecture, as well as privacy safeguards and end-to-end latency from signal acquisition to adaptive feedback.”

**Reviewer #1, Comment #4:** Generic labels like 'distraction' limit cross study comparability. Suggest mentioning a future taxonomy of IVR learning tasks in the Conclusion’s 'Future research' part.

**Author response:** Thank you for raising an important point about construct comparability across IVR learning and training studies. Generic labels such as “distraction,” “attention,” “concentration,” “immersion,” and “cognitive load” may appear similar across studies, but they are often defined through different task settings, behavioral contexts, experimental manipulations, and label sources. We recognize that, although the original manuscript discussed the heterogeneity of target constructs and label sources, it did not explicitly identify the need for a task-oriented taxonomy as a future research direction.

In response to this comment, we added the following sentence to the Conclusion (lines 539-544):

“Future research should also develop a task- and context-sensitive taxonomy of IVR learning and training scenarios and target constructs, so that generic labels such as distraction, attention, concentration, immersion, and cognitive load can be defined in relation to specific task demands, learner actions, experimental manipulations, and label sources, thereby improving cross-study comparability.”

**Reviewer #1, Comment #5:** Last but not least, Please reformat the manuscript carefully: centre figures, correct reference numbers in tables (e.g., Table 1 and Table 4 formatting), and fix author affiliation marks on the title page. Current alignment and table reference presentation are not up to journal standard; a thorough formatting check is needed.

**Author response:** We have reformatted the manuscript using the journal’s template and conducted a full formatting check. Figures were centered, author affiliation marks on the title page were corrected, and the formatting of Tables 1 and 4 was revised. We also checked the reference numbers presented in the tables and corrected formatting inconsistencies in captions, table notes, and reference presentation.

**Reviewer #2, Comment #1:** Please proofread references and subsection numbering

**Author response:** We proofread and corrected the references, in-text citations, table citations, and subsection numbering throughout the manuscript.

**Reviewer #2, Comment #2:** Some conclusions are written like settled findings but are based on very small subgroups. With only 19 studies the language needs to be more careful throughout.

**Author response:** Thank you for the comment, which highlights the need to align the strength of our wording with the size and nature of the evidence base. We have revised the manuscript to make the language more cautious throughout. In particular, we replaced stronger expressions such as “the field has established,” “dominant,” with more bounded phrasing such as “the included studies collectively outline,” “in this review sample,” and “the findings suggest.” We also added clearer qualifiers when discussing small subgroups, especially for multimodal fusion strategies, validation designs, and deployment readiness. These revisions preserve the descriptive findings of the scoping review while making the interpretation more proportionate to the sample size.

In the Abstract, we made the following revision:

“In the 19 included studies, eye-tracking data (ETD, 13/19) and central nervous system signals (CNS, 11/19) were the most frequently reported modalities. CNS+ETD was the most frequently reported dual-modality configuration. Peripheral physiological signals (PPS, 3/19) were less frequently represented, and studies using electrodermal activity (EDA) were especially limited within the included corpus. The included studies covered the main stages of acquisition, preprocessing, feature engineering and representation learning, fusion, and analysis and modeling.”

In the Results section:

(lines 278-280)

“Overall, within the included studies, single-modality and dual-modality designs were more common than designs collecting all three physiological signal categories.”

(lines 292-294)

“In contrast, preprocessing and artifact-management procedures were reported somewhat more often, and some modality-specific reporting patterns were observed in the included studies.”

(lines 330-333)

“Under the early/intermediate/late classification framework in multimodal fusion research [74, 75], early fusion was the most frequently reported fusion strategy among the 12 fusion studies (11/12), whereas late fusion appeared in 1 study and no included study reported intermediate or hybrid fusion.”

(lines 346-351)

“Among the 8 multimodal studies, 5 used only traditional machine learning, while 3 reported both ML and DL, mainly in CNS+ETD studies. In this review sample, studies reporting both ML and DL were more frequently observed in multimodal studies than in single-modality studies, although this pattern should be interpreted cautiously because of the small subgroup sizes.”

(lines 353-354)

“In the included studies, classification was the most frequently reported AI task, appearing in 17 studies (89.5%).”

(lines 362-363)

“Within this review sample, work on continuous state estimation, forward-looking prediction, and anomaly detection was less frequently represented.”

(lines 388-391)

“In this review sample, AI-based physiological signal analysis was mainly reported in offline discrimination settings, and evidence for real-time, continuous, closed-loop IVR adaptation under strict generalization testing was limited.”

In the Discussion section:

(lines 402-403)

“In terms of modality coverage, ETD (13/19) and CNS (11/19) were the two most frequently reported sources in the included IVR learning studies.”

(lines 420-421)

“This pattern may indicate an emerging interest in multi-source modeling within the included studies.”

(lines 465-466)

“At the fusion level, early fusion was the most frequently reported strategy among the 12 fusion studies.”

(lines 475-476)

“In terms of validation design, label sources, and deployment readiness, the included evidence was concentrated mainly in offline classification studies.”

(lines 491-493)

“From the overall reporting pattern in the included studies, one recurring issue was not simply whether AI was used, but whether the sensing system was described clearly enough to support reproduction, comparison, and deployment.”

In the Conclusion section:

(lines 517-519)

“The findings suggest that, in the included literature, progress in AI-based physiological signal analysis for IVR learning depends not only on model performance, but also on the quality of sensing-system reporting and the clarity of validation design.”

(lines 533-535)

“Overall, the included studies collectively outline an initial technical pathway. However, within this review sample, evidence for reproducible, verifiable, and deployment-oriented real-time closed-loop IVR physiological sensing systems remained limited.”

In the Limitations section:

(lines 555-556)

“The 19 included studies still provide an initial overview of several technical paths observed in the current literature, although this overview should not be considered exhaustive.”

**Reviewer #2, Comment #3:** No prospective registration was done. A short explanation of why should be added, along with confirmation that the criteria were set before screening started.

**Author response:** Thank you for this helpful suggestion on review transparency. The review was not prospectively registered because it was planned as an exploratory scoping review of technical configurations, reporting practices, and evidence gaps in an emerging interdisciplinary area. It was not designed to estimate intervention effects, compare effect sizes, or conduct a quantitative synthesis.

To improve transparency, we revised the Materials and Methods section to explain the absence of prospective registration and to state more clearly that the review questions, eligibility criteria, information sources, search strategy, and data-extraction framework were established before screening began. The added text reads (lines 125-132):

“No public protocol was prospectively registered for this review. The study was designed as an exploratory scoping review to map technical configurations, reporting practices, and evidence gaps in an emerging interdisciplinary area, rather than to estimate intervention effects or conduct a quantitative synthesis. Before screening began, the review questions, eligibility criteria, information sources, search strategy, and data-extraction framework were defined. These procedures were then applied consistently during title, abstract, and full-text screening, as well as during data extraction.”

**Reviewer #2, Comment #4:** A few parts of the discussion describe things as clear trends when the numbers are actually quite small. Needs to be toned down in places.

**Author response:**

Thank you for this careful observation. We revised the Discussion throughout to use more cautious and scope-bounded language. In particular, we avoided phrasing that might imply clear field-level trends when the underlying evidence came from small subgroups. This revision was made as part of the broader language revisions described in our response to Reviewer #2, Comment #2, and we paid particular attention to the Discussion section when making these changes. We toned down trend-like statements in the Discussion and revised the wording to better reflect the small size of the evidence base. Stronger expressions such as 'dominant,' 'current evidence is dominated,' 'the field is moving toward,' and 'the main issue is' were replaced with more cautious wording such as 'most frequently reported,' 'the included evidence was concentrated mainly in,' 'may indicate an emerging interest,' and 'one recurring issue was.' For readability, the corresponding revised manuscript text is presented under Reviewer #2, Comment #2, where the full set of scope-bounded language revisions is listed.
